# Assessment of reconstruction accuracy for under-sampled 31P-MRS data using compressed sensing and a low rank Hankel matrix completion approach

**DOI:** 10.3389/fendo.2025.1581328

**Published:** 2025-06-17

**Authors:** Jossian A. García, Michael D. Noseworthy, Alejandro Santos-Díaz

**Affiliations:** ^1^ Tecnologico de Monterrey, School of Engineering and Sciences, Mexico City, Mexico; ^2^ Electrical and Computer Engineering, McMaster University, Hamilton, ON, Canada

**Keywords:** ^31^P-MRS, energy metabolism, compressed sensing, low rank Hankel matrix completion, reconstruction accuracy

## Abstract

Phosphorus magnetic resonance spectroscopy and spectroscopic imaging (^31^P-MRS/MRSI) are techniques to evaluate energy metabolism *in vivo*, they are capable of measuring metabolites such as phosphocreatine and inorganic phosphate in muscle and brain tissue. Despite their capability, these techniques are not very often used in clinical settings due to the long acquisition times required. In recent years, compressed sensing has been widely used as an acceleration method for MRI signal acquisition and translated to MRS. In order to use it, one of the main criteria states that the aliasing resulting from the undersampling scheme must be incoherent, which is achieved using a pseudo-random sampling strategy. However, when a set of pseudo-random sampling patterns are applied for the same acceleration factor, there is significant variability in the quality of the reconstructed signal. We present an evaluation of the influence of the undersampling pattern in the quality of the signal reconstruction through a series of experiments in ^31^P-MRS data using the low rank Hankel matrix completion as the reconstruction method. Our results demonstrate that the reconstruction accuracy is heavily influenced by the selection of specific samples rather than the undersampling factor. Furthermore, the noise level in the signal has a more pronounced impact on reconstruction quality. Additionally, reconstruction accuracy is significantly correlated with the density of samples collected at early sampling times, making it possible to set large time values to zero without producing any statistical difference in the error distribution means for some cases.

## 1 Introduction

Phosphorous Magnetic Resonance Spectroscopy (^31^P-MRS) provides valuable information about energy metabolism, membrane degradation and pH *in vivo*. ^31^P-MRS is able of tracking metabolites such as phosphocreatine (PCr), adenosine triphosphate (ATP), and inorganic phosphate (Pi), information useful in the medical study of many disorders related to bioenergetic abnormalities in the brain (1). Despite the medical utility, ^31^P-MRS is not often used in clinical settings due to a number of limitations. First, the low concentration of metabolites, which leads to a low signal-to-noise ratio (SNR). Second, a low gyromagnetic ratio which causes low nucleus sensitivity, and third, the relatively short T_2_ relaxation times of some metabolites that induce ^31^P-MRS acquisition to take longer times.

Some efforts have been made to translate common acceleration methods in magnetic resonance imaging (MRI) to ^31^P-MRS, for instance echo planar spectroscopic imaging (EPSI) (2, 3), spiral trajectories (4), and variations of compressed sensing (CS) (5, 6). Among these, CS has emerged as an effective acceleration method by reconstructing undersampled signals. In order to apply this technique there are three main requirements that need to be met (7): (1) the data have a sparse representation in a transformed domain; (2) the aliasing generated due to the subsampling scheme is incoherent (noise-like); and (3) a non-linear reconstruction method is to be used in order to enforce consistency with the measurements and sparsity of the data. The problem can be formulated as reconstructing the spectrum *x* from the equation $F_{u}x=\hat{y}$ , where $\hat{y}$ represents the acquired data points, with zeros filling the positions where no sampling occurs. Here, F_u_ denotes the undersampled inverse Fourier transform operator. CS approach solve this problem by finding the sparsest solution for the measurements $\hat{y}$ , this is achieved by minimizing Equation 1. The claim of a sparse spectrum means that only few frequencies give rise to true peaks while the rest of the spectra contain only baseline noise (8).


(1) $$\text{argmin}\;\left||x{\|}_{1}\;\text{subjectto}\;\right||F_{u}x-\hat{y}{\|}_{2}<\epsilon$$

Here, L1 norm $\|x{\|}_{1}=\sum_{i}|x_{i}|$ and by minimizing it we promote sparsity while $\|F_{u}x-\hat{y}{\|}_{2}<∊\;$ enforces data consistency.

However, the low rank Hankel Matrix completion approach has demonstrated superior performance in the context of ^31^P-MRS signals compared to traditional CS (5, 9). This method addresses the problem by operating in the time domain. The free induction decay (FID) signal *y* is recovered by solving the optimization problem presented in Equation 2.


(2) $$\underset{y}{\min}\;\;\|Ry{\|}_{*}+\frac{\lambda }{2}\|\hat{y}-Uy{\|}_{2}^{2}$$

Where *y* is the complete FID to be estimated, *R* is an operator that converts *y* into the Hankel matrix showed in Equation 3:


(3) $$H\left(y\right)=Ry=\left[\begin{array}{ccccc} f_{1} & f_{2} & f_{3} & \ldots & f_{Q}\\ f_{2} & f_{3} & f_{4} & \ldots & f_{Q+1}\\ \vdots & \vdots & \vdots & \ddots & \vdots \\ f_{n-Q} & f_{n-Q+1} & f_{n-Q+2} & \ldots & f_{n-1}\\ f_{n-Q+1} & f_{n-Q+2} & f_{n-Q+3} & \ldots & f_{n} \end{array}\right]$$

U is an undersampling operator, *λ* is the data consistency parameter, and the nuclear norm is denoted as ||*…*||_∗_. It has been proven that the rank of the Hankel matrix is equal to the number of decaying exponentials in the FID, so the low rank approach reconstructs a spectrum with the least number of spectral peaks. Finally, the solution to Equation 2 can be found using the alternating direction minimization method (ADMM) (8).

To implement the subsampling schemes, a novel pulse sequence that combines a flyback EPSI readout with compressed sensing (fidepsiCS) has been used (9–11). The fidepsiCS sequence has the characteristic that when applying CS, the subsampling is performed in the temporal dimension due to gradient blips incorporated in the phase encoding direction. The use of the fidepsiCS sequence can reduce acquisition times considerably when compared to traditional chemical shift imaging and EPSI (9, 11).

Recently, our group showed that when a series of different undersampling patterns are applied for the same acceleration factor over ^31^P-MRS data, there is significant variability in the quality of the reconstructed signal (10). Furthermore, we observed that the performance of the reconstruction depends almost entirely on the samples selected by the pseudo-random pattern. In this work we expand the research by presenting an evaluation of the influence of a Non-Uniform Sampling (NUS) scheme in the quality of the signal reconstruction throughout a series of experiments over simulated and *in vivo* ^31^P-MRS data using the low rank Hankel matrix completion approach originally presented by Qu et al. (8).

## 2 Materials and methods

### 2.1 ^31^P-MRS data simulation

**Figure 1:**
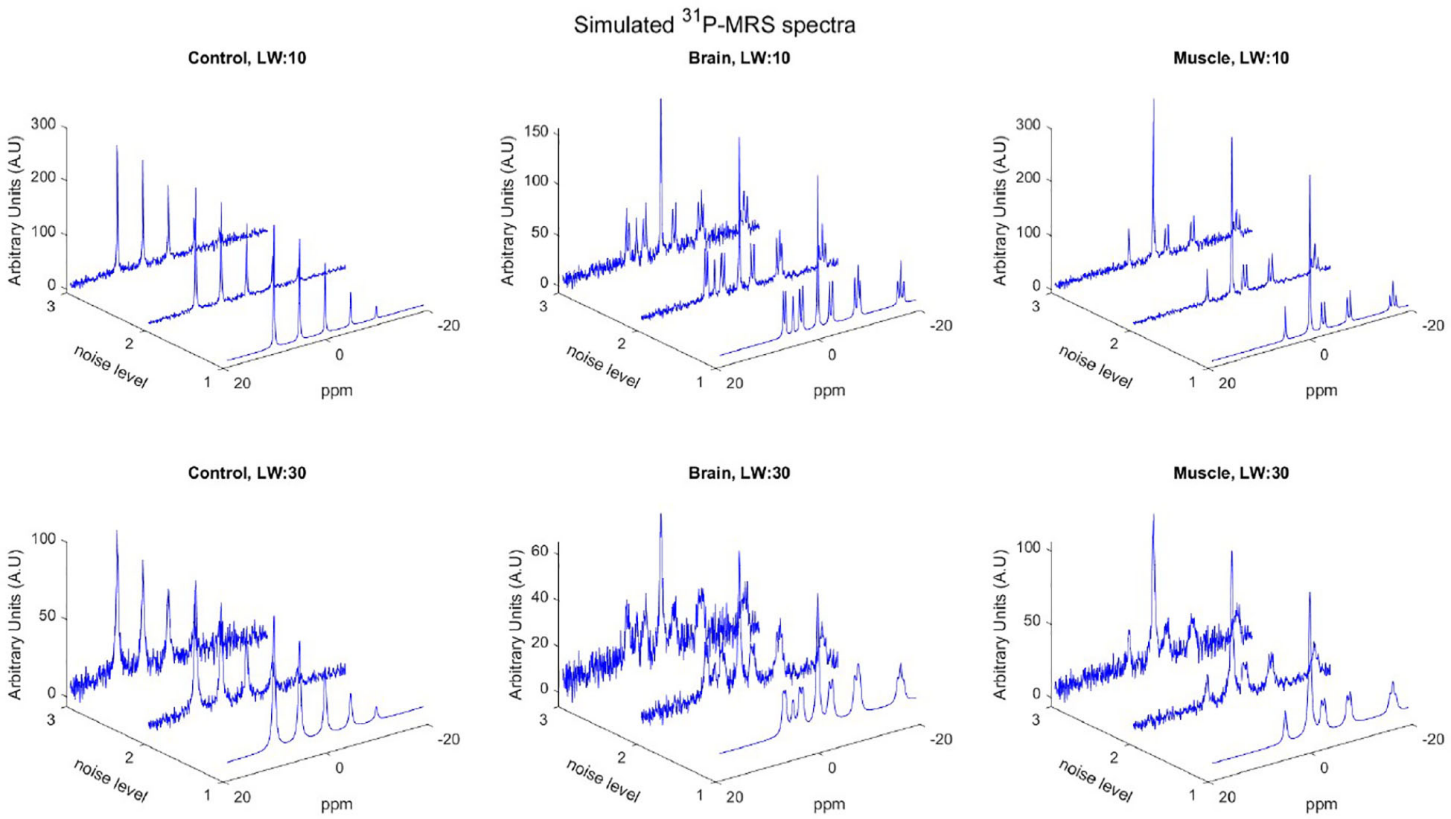
Three types of ^31^P-MRS simulated spectra (control, brain and muscle) including different noise levels, as well as two different linewidths: top row corresponds to 10Hz and bottom row to 30 Hz.

Simulated spectra were generated using a modified version of the FID-A toolbox, adapted for the simulation of ^31^P-MRS (12). All simulations were performed assuming a magnetic field strength of 3T and a spectral bandwidth of 2000 Hz. To generate a robust and comprehensive dataset, various conditions were considered, including linewidths of 10 Hz and 30 Hz, three levels of Gaussian noise (with standard deviations of *s* = 0, *s* = 2.5, and *s* = 5), and three distinct scenarios based on the metabolite peaks present in each spectrum as shown in **Figure 1**. First, a control simulation was generated, containing five amplitude peaks at -10, -5, 0, 5, and 10 ppm. Second, simulations of human skeletal muscle tissue included PCr, Pi, and ATP metabolites. Third, brain tissue simulations incorporated PCr, Pi, ATP, glycerophosphocholine (GPC), glycerophosphoethanolamine (GPE), phosphoethanolamine (PE), nicotinamide adenine dinucleotide (NAD), and phosphocholine (PC). Peak amplitudes were assigned according to metabolite peak ratios observed *in vivo* acquisitions. Control, brain, and skeletal muscle simulations each contained 512 data points. Additionally, simulations with 1024 data points were generated for control and brain conditions.

### 2.2 ^31^P-MRS data acquisition

Data were collected using a 60 cm bore 3T GE MR750 (GE Healthcare, Milwaukee, WI) scanner (50 mT/m amplitude and 200 T/m/s slew rate gradient system). Phantom acquisitions were performed on a custom-built spherical phantom containing 25 mmol/L and 10 mmol/L concentrations of sodium phosphate (P1) and phosphocreatine disodium salt (P2) respectively, using an in house designed/built 24 cm diameter quadrature birdcage coil tuned to 51.720 MHz. On the other hand, *in vivo* ^31^P-MRS data were acquired from eight healthy volunteers (all males, 24 ± 6 years of age) who provided informed consent to participate in this study. Five brain signals were acquired from the parietal lobe of five of the volunteers using a 12.7 cm diameter home designed/built surface coil (51.705 MHz) and a single volume pulse-acquire sequence, squared excitation pulse (0.5 ms, 60°flip angle), 2000 Hz spectral bandwidth, 512 points, 128 averages. Three skeletal muscle signals were collected from the gastrocnemius in the calf muscles of the remaining three participants using an in-house designed/built ^31^P-tuned (51.705 MHz), 7.62-cm-diameter surface coil. Radiofrequency excitation was calibrated to achieve a 90°flip angle at approximately 3.5 cm from the coil’s center. All procedures were conducted in compliance with the ethical standards of our institutional research ethics board, following the principles outlined in the 1964 Helsinki Declaration on human ethics.

### 2.3 Undersampling and reconstruction

FID signals were retrospectively undersampled using pseudo-random uniform distributions. For 1024samples signals, undersampling factors (USF) of x2 (512 out of 1024), x3 (341 out of 1024) and x4 (256 out of 1024) samples were implemented. For 512-sample acquisitions, acceleration factors resulted in 256, 170 and 128 out of 512 sample signals, respectively. A total of 1000 different schemes per USF were generated through a Monte Carlo-like simulation. In addition, an exponentially decaying Non-Uniform Sampling (NUS) approach, based on the NUSSAMPLER program from the MDDNMR package (13), was also used to generate sampling patterns biased toward the origin.

Compressed Sensing was applied over each type of FID signal using the sets of undersampled schemes previously generated. The low rank Hankel matrix completion was used as the reconstruction method, specifically, the ADMM approach presented by Qu et al (8),. The value of the trade-off was set as *λ* = 10^3^ since the authors stated that results of calculations are not sensitive to the setting of *λ*. On the other hand, the ADMM requires another parameter which influence the reconstruction accuracy, the Q value, which was set to *Q* = 256 for 512-sample signals and *Q* = 512 for 1024 sample signals. The low rank calculations were performed using Matlab (The MathWorks Inc.) on a laptop computer with Intel Core i5-1155G7 with 4 Cores, 2.5 GHz CPU and 16 GB RAM. Single reconstructions of skeletal muscle signals using USFs of x4, x3, x2 took 14.18, 6.49 and 2.47 seconds respectively.

### 2.4 Statistical analysis

A series of statistical experiments were executed to find patterns and explain the behaviors in the data. The root mean square error (RMSE) was the metric used to measure the performance of the reconstruction compared to the original spectra. The statistical analysis included: RMSE distribution plots, Lilliefors tests, correlation matrices, violin plots, Kruskal-Wallis tests. Violin plots were useful to visualize general behavior of error in different scenarios, while RMSE distribution and Lilliefors test were used to determine if the error followed a Gaussian distribution.

**Figure 2:**
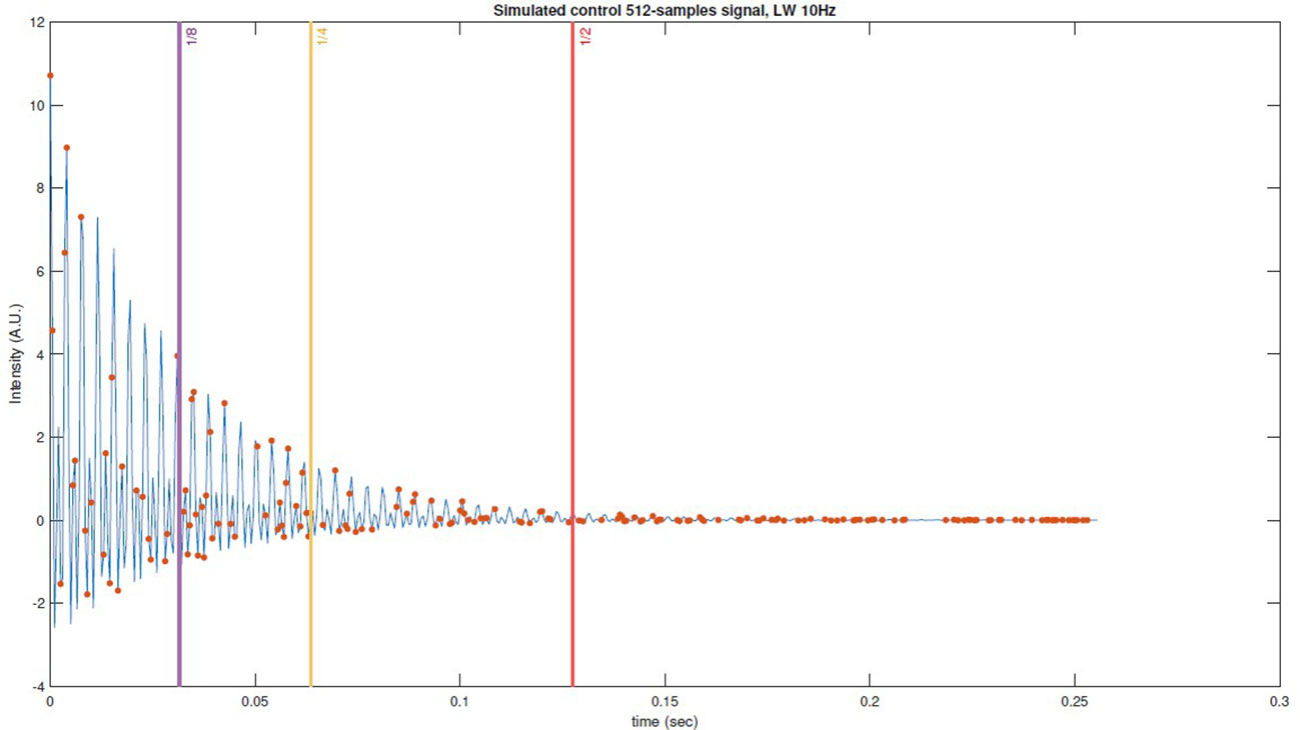
Example of simulated FID control data with thresholds marked with vertical lines, pseudorandom subsampling scheme (USF x2) with red dots and the original signal in color blue.

In addition, we used the number of samples taken per octile of the signal as a metric to assess the impact of sample density at the onset of the pseudo-random acquisition. This metric was then used to compute Pearson correlation coefficients in relation to the RMSE of the reconstructions. Subsequently, different thresholds were set at the midpoint, the fourth, and the eighth part of the FID signal. Time values exceeding these thresholds (as shown in **Figure 2**) were set to zero, and the reconstructions were performed again. The Kruskal-Wallis test was conducted to determine whether truncation led to statistically significant differences among the error distributions means.

## 3 Results

**Figure 3:**
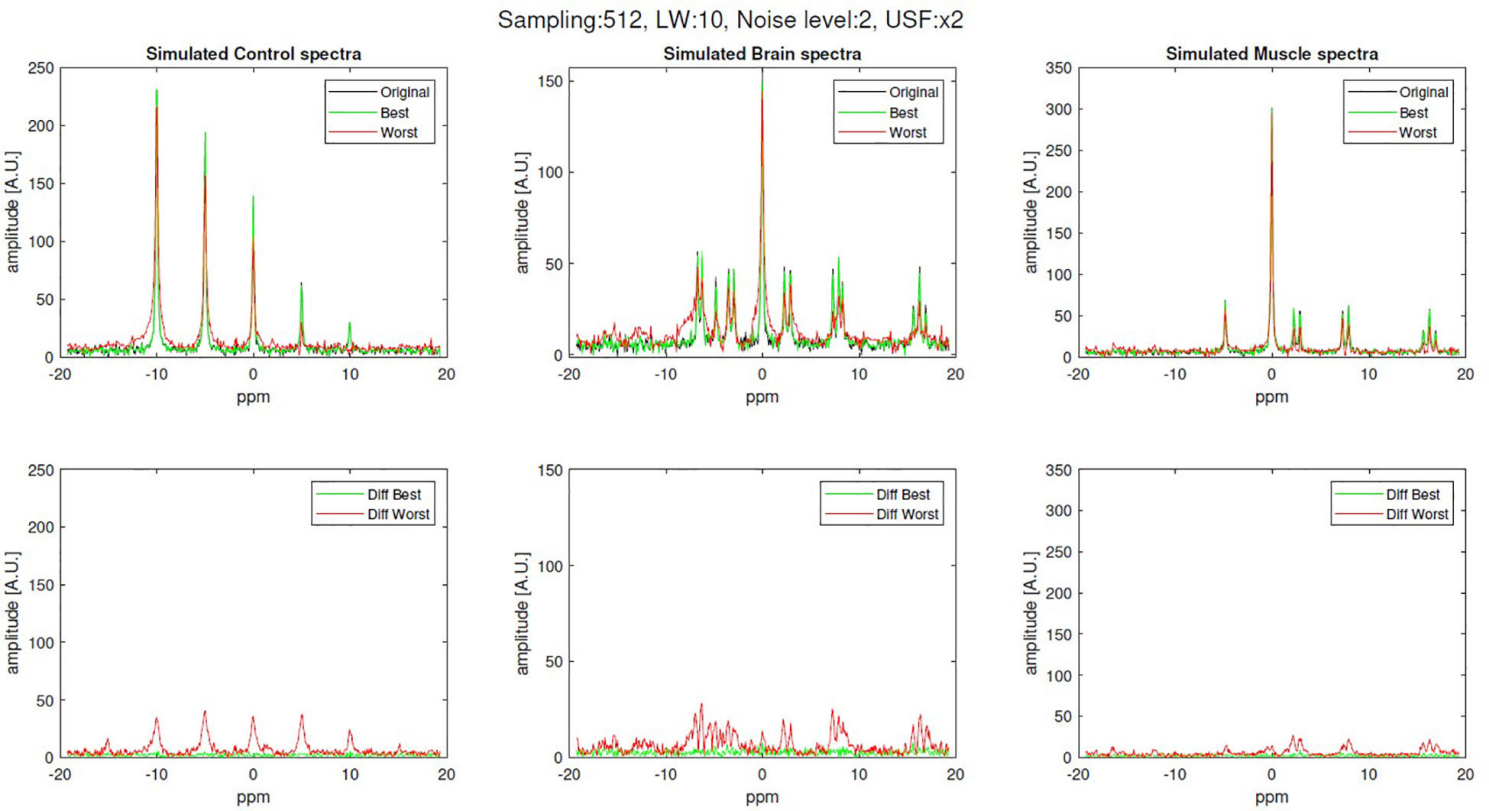
Examples of original (fully sampled), best and worst reconstruction (512-samples, x2 USF, noise level 2 and linewidth of 10 Hz) for brain and skeletal muscle simulations along with their difference.

**Figure 3** shows examples of the best and worst reconstructed spectra for control, brain and skeletal muscle simulated sets, as well as their residual against the fully sampled (original) signal. These reconstructed spectra correspond to 512-samples simulations with noise level of 2 and USF x2.

The Pearson correlation coefficients obtained (**Supplementary Tables S1, S2**) indicated a moderate to strong correlation for both 512 and 1024 samples simulations. Notably, these coefficients tended to increase as the USF became more aggressive. This suggests that reconstructions with lower errors tend to have a higher density of samples taken at the onset of the FID.

**Figure 4:**
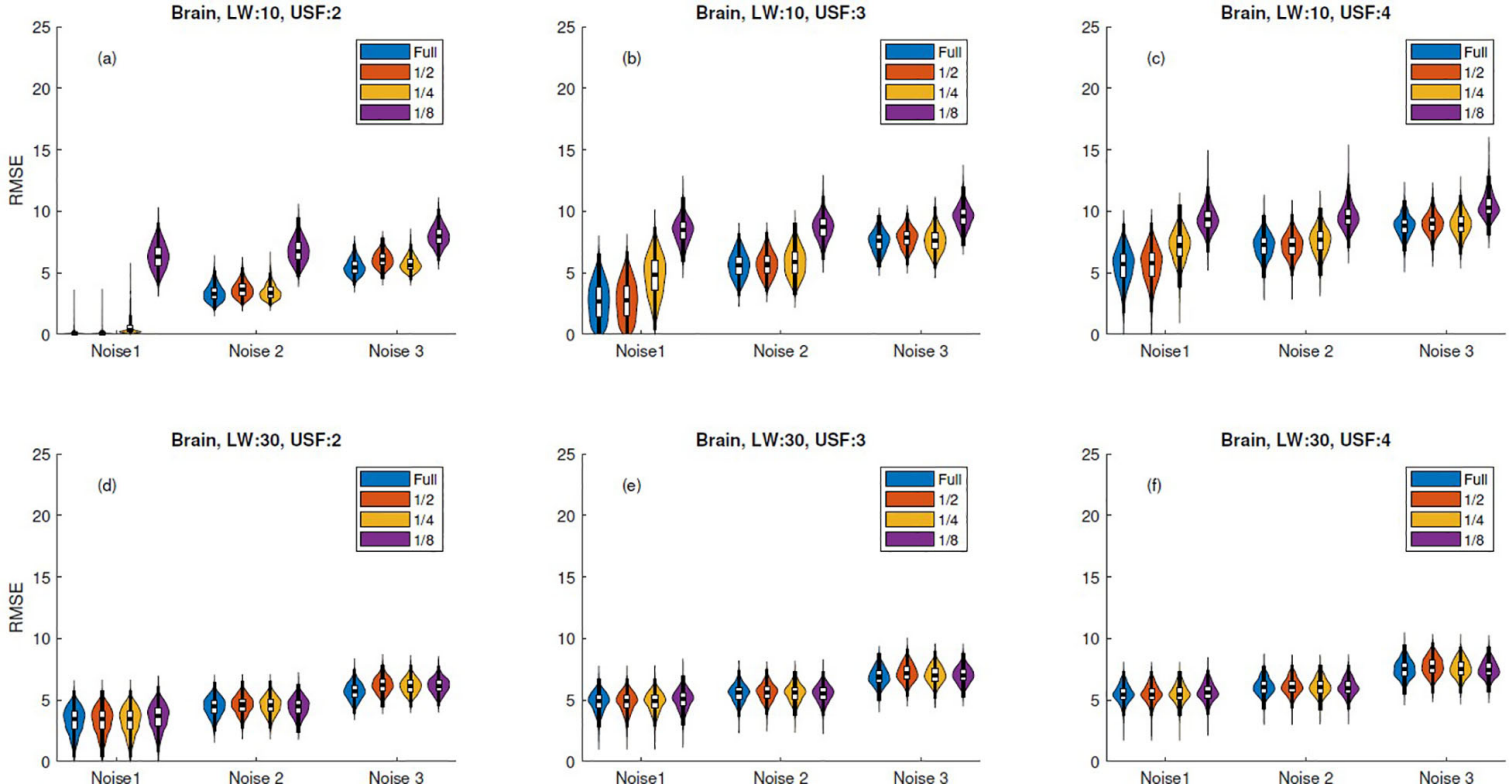
RMSE violin plots for 512-sample brain simulation reconstructions with truncation. Columns correspond to undersampling factors (USF = 2, 3, 4), and rows to linewidths (LW = 10 Hz, 30 Hz). Subplots **(a–f)** represent all LW–USF combinations: **(a–c)** for LW = 10 Hz and **(d–f)** for LW = 30 Hz. Each violin subplot shows RMSE distribution across three noise levels and truncation levels ("Full", 1/2, 1/4, 1/8), where "Full" corresponds to no truncation.

Regarding the truncation experiments presented in Section 2.4, **Figure 4**, **Supplementary Figures S1, S5** illustrate that truncation had minimal impact on the RMSE distribution variance for signals with 512 samples and a linewidth of 30 Hz. However, for reconstructions with lower linewidths, the error distribution variance showed a notable increase, particularly in the case of one-eighth truncation. In contrast, when truncation was applied to signals with 1024 samples, the RMSE distribution variance remained barely affected, as shown in **Supplementary Figures S2, S4**.

### 3.1 *In vivo* ^31^P-MRS data results

**Figure 5:**
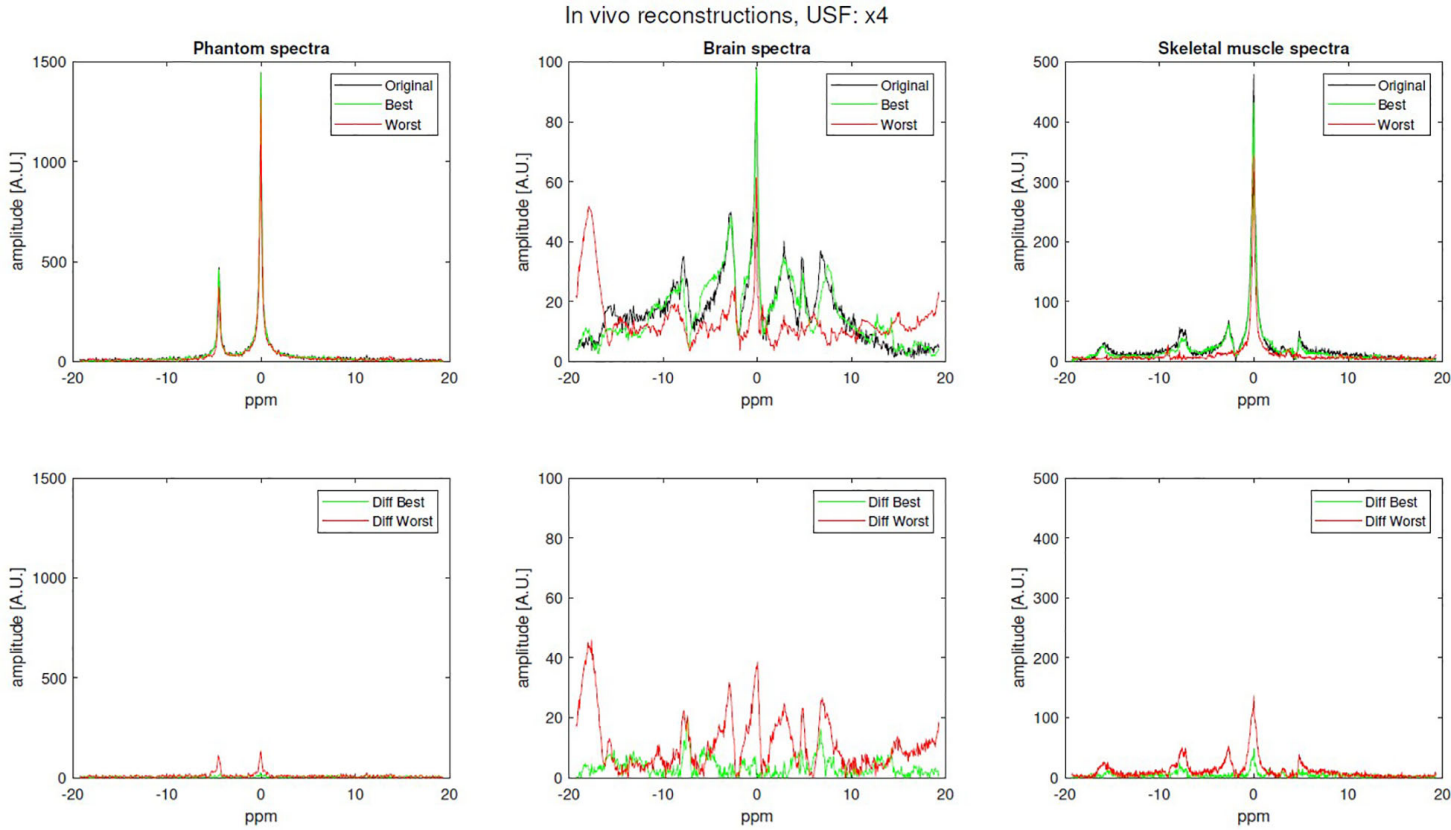
Examples of original (fully sampled), best and worst reconstruction (x4 USF) for phantom, brain and skeletal-muscle along with their difference.

In total, eight different *in vivo* ^31^P-MRS signals were acquired and analyzed: four brain signals with 512 sampled points, one brain signal with 1024 sampled points and three skeletal muscle signals with 1024 sampled points. Examples of the best and worst reconstructed spectra (x4 USF) for phantom, brain, and skeletal muscle are shown in **Figure 5**.

**Supplementary Figure S6** illustrates the reconstruction error distributions for Phantom signals, while **Supplementary Figure S7** presents examples of reconstructed spectra. The USF had a small impact on the reconstruction error distribution variability. However, truncation experiments resulted in a clear increase in RMSE variability, highlighting the sensitivity of reconstructions to early signal truncation.

**Figure 6:**
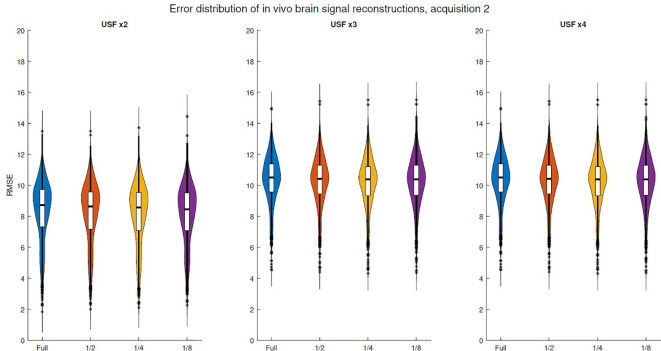
RMSE violin plots corresponding to *in vivo* brain signal reconstructions for the second acquisition. USF of x2: using 256 out of 512. x3: using 170 out of 512. x4: using 128 out of 512.

**Figure 6** and **Supplementary Figures S8–S11** illustrate the error distributions for truncation experiments across five *in vivo* brain acquisitions. All acquisitions exhibited a similar trend, as truncation did not lead to a noticeable increase in the distribution means even with maximum truncation. However, the reconstruction error for signals with 1024 samples (acquisition 1) showed lower variance. The corresponding plots for skeletal muscle acquisitions are shown in **Supplementary Figures S12–S14**. Similar to brain signals, the error variability for skeletal muscle did not exhibit an evident increase in their means, indicating that truncation had limited impact on reconstruction errors.

**Figure 7:**
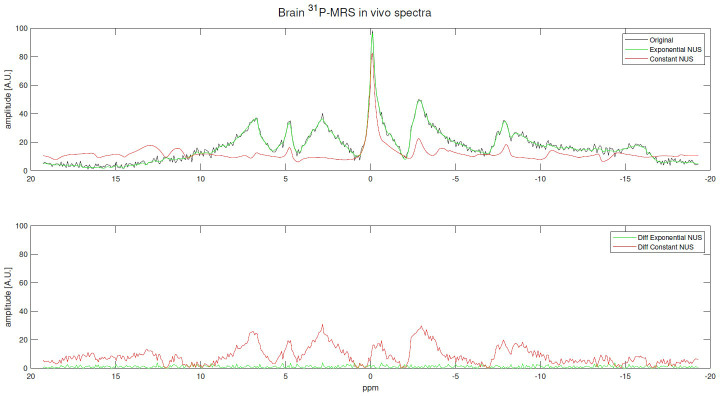
Examples of *in vivo* ^31^P MRS brain signal reconstructions using an exponentially NUS and constant NUS along with their differences. The original is the fully sampled (512 points) signal and both NUS patterns with USF x4: 120 out of 512 points.

**Figure 7**, **Supplementary Figure S16** present examples of reconstructions for *in vivo* ^31^P MRS brain and skeletal muscle spectra recovered from exponentially and constant NUS signal reconstruction, both with USF x4.

## 4 Discussion

The results in **Supplementary Tables S1, S2** highlight the crucial role of early-time samples in reconstruction accuracy, as the correlation coefficients are significant in most cases. Overall, a more aggressive USF generally leads to an increase in Pearson’s correlation coefficient, particularly when reconstructing the sparsest signals with 512 samples.

**Supplementary Figures S1–S5** illustrate that signal reconstructions with higher linewidth values are minimally affected by truncation. This can be attributed to the fact that signals with lower linewidth exhibit a slower exponential decay, causing their values to take longer to approach zero. Consequently, truncation excludes significant values, leading to an increase in the RMSE mean under aggressive limits. However, reconstruction errors for 1024-sample simulations appear to be more robust to aggressive truncation limits, even in cases with a small linewidth. Since not all error distributions are normal, a Kruskal-Wallis test was performed on each RMSE reconstruction distribution against its corresponding truncations, the maximum truncation of $\frac{1}{8}$ was not considered in the test as it provoked a profound increase in variability in some of the distributions. Considering only linewidth of 30 Hz, analysis showed that for ideal signals (noise level 1) there was no statistical difference between fully undersampled reconstructions and those using truncation at one-fourth of the signal, with *p* = 0.05 for both 512 and 1024-sample spectra reconstructions. Regarding linewidth = 10 Hz, truncation over reconstructions only provoke a slight variation in the RMSE distribution.

On the other hand, to explain the error increment observed in **Supplementary Figure S6**, it is important to analyze two factors regarding phantom experiments and truncation: 1) Brain and skeletal muscle chemical environments provokes different relaxation times and change the metabolite peaks amplitude in the spectrum. 2) As Kazimierczuk et al. and Rovnyak et al. concluded in (14) and (15) respectively, an extreme signal truncation can discard too much information at long times and deteriorates the spectra resolution. 3) The RMSE metric aggressively penalizes larger errors due to squaring. As illustrated in **Supplementary Figure S7**, the phantom spectrum contains peaks with significantly higher amplitudes compared to brain and skeletal muscle spectra. Greater truncation results in increased resolution loss, which in turn increases error.

Violin plots of *in vivo* error spectra reconstructions exhibit a behavior similar to that observed in simulated spectral reconstructions. For instance, the *in vivo* brain and skeletal muscle spectra analyzed in **Figure 6**, **Supplementary Figure S13**, respectively, show no significant variation in distributions when comparing the fully undersampled signal with a truncation threshold at the middle (1/2), as confirmed by the Kruskal-Wallis test, even in schemes with USF ×4. Moreover, in the case of USF x3 in **Figure 6**, all distribution means can be considered equivalent. It is important to notice that although *in vivo* error distribution means are higher than in simulations, the effect of the truncation is more similar to simulations with linewidth of 30 Hz, where even the higher level of truncation does not produce a relevant increase in the error distributions. The above indicate that simulations using linewidth of 30 Hz imitate better the real properties of the *in vivo* FID acquisitions.

Some studies in nuclear magnetic resonance (NMR) have shown that selecting samples randomly, following an exponentially decaying distribution with a time constant *T*_2_, results in improved performance when using methods such as traditional compressed sensing (CS) and minimum entropy reconstruction (14, 15). It was observed that to further enhance the SNR, it is necessary to apply an extreme bias toward early-time sampling (by reducing the *T_s_* value), but without exceeding *T_s_ < T*_2_ in order to preserve spectral resolution. To compare the improvement in reconstruction accuracy using this exponentially decaying probability distribution, we performed a single reconstruction for each type of signal and for each different undersampling factor (USF). A *T*_2_ value of 50 ms was selected, which is a relatively long relaxation time for ^31^P compounds at 3T.

The RMSE of reconstructions using exponentially biased NUS were, in most of the cases, better than the best value reached by the Monte Carlo simulation of 1000 ^31^P-MRS reconstructions. Furthermore, experiments using *in vivo* ^31^P-MRS data presented a RMSE up to 85.84% lower using an exponential NUS compared to constant NUS, and a 60.40% lower error for the worst case of the ^31^P-MRS brain spectrum. Regarding skeletal muscle spectra, reconstructions showed a RMSE up to 74.12% lower and a minimal enhancement of 25.8%. Finally, selecting an undersampling pattern that prioritizes samples near the origin, as done with exponentially biased NUS, yields better results. However, this approach may not be universally applicable to all types of MRS signals as the challenge falls into the realm of pulse sequence design.

One important limitation of this study was that the evaluation of reconstruction performance was based on global spectral metrics, such as RMSE and overall spectral SNR, without a detailed analysis of individual metabolite peaks. It is well known that CS can differentially impact spectral features depending on their contrast-to-noise ratio (CNR), potentially leading to selective attenuation of low-amplitude peaks during iterative denoising. Therefore, future work will require a more targeted, metabolite-by-metabolite quantitative assessment to more accurately validate the fidelity of the proposed method in reconstructing individual spectral components.

## 5 Conclusion

In conclusion, this study performed an exhaustive analysis of the reconstruction accuracy over control, brain and skeletal muscle simulated ^31^P-MRS data with two different linewidths of 10 and 30Hz. Additionally, three levels of noise were added to FID signals in order to generate a coarse dataset useful to analyze in deep the effects of CS using low rank Hankel matrix completion as the reconstruction method. Data was retrospectively undersampled using USFs of x2, x3 and x4 for 1024 and 512-samples signal acquisitions. *In vivo* acquisition corresponded to 4 brain signals with 512 spectral points, one brain signal with 1024 spectral points and 3 skeletal muscle signals with 1024 spectral points. Our results show that the reconstruction accuracy is highly dependent on the selected samples. Our main contributions can be expressed in the following:

- Spectra with higher linewidth were minimally affected by setting large time values to zero.
- Reconstruction accuracy is significantly correlated with the density of samples taken at early times.
- According to *in vivo* results, it is possible to establish a threshold at the middle and set large time values to zero without producing a statistical difference in the error distribution means, even in the schemes with USF x4.
- The use of exponentially NUS meant a reconstruction accuracy enhancement up to 85.84% and 74.12% for 512-samples ^31^P-MRS brain and 1024-samples ^31^P-MRS skeletal muscle data respectively.
- The increase of the sampling density near to the origin is especially useful in 512-samples signals where it showed the best reconstruction results.

Despite the advantages of exponentially decay NUS, studies have been concentrated in the analysis of synthetic biased signals and no effort has been made in the study of pulse sequences to acquire a FID signal with these characteristics. Further analysis should be focused on the development of pulse sequences.

## Data Availability

Data is available upon request. Requests to access these datasets should be directed to alejandro.santos@tec.mx.
