# Supplementary material for: Assessment of reconstruction accuracy for under-sampled 31P-MRS data using compressed sensing and a low rank Hankel matrix completion approach
Source: Front Endocrinol (Lausanne). 2025 Jun 17;16:1581328. doi: 10.3389/fendo.2025.1581328 (PMC12208845; doi:10.3389/fendo.2025.1581328)
Supplement: Supplementary file 1 [file DataSheet1.pdf]

Supplementary Material

1 SUPPLEMENTARY TABLES AND FIGURES

1.1 Tables

Table S1. Pearson correlation coefficients (number of samples in the first octile vs RMSE) for simulated 512-samples signal reconstructions.

	Noise	LW:10			LW:30		
		x2	x3	x4	x2	x3	x4
Control	1	-0.10	-0.14	-0.28	-0.47	-0.56	-0.59
	2	-0.38	-0.36	-0.43	-0.46	-0.51	-0.55
	3	-0.42	-0.40	-0.48	-0.42	-0.47	-0.55
Brain	1	-0.41	-0.52	-0.51	-0.56	-0.53	-0.56
	2	-0.61	-0.52	-0.56	-0.49	-0.50	-0.54
	3	-0.56	-0.55	-0.58	-0.44	-0.48	-0.54
Muscle	1	-0.36	-0.45	-0.56	-0.47	-0.56	-0.59
	2	-0.60	-0.54	-0.59	-0.46	-0.51	-0.56
	3	-0.53	-0.57	-0.60	-0.42	-0.47	-0.55

Table S2. Pearson correlation coefficients (number of samples in the first octile vs RMSE) for simulated 1024-samples signal reconstructions.

	Noise	LW:10			LW:30		
		x2	x3	x4	x2	x3	x4
Control	1	-0.44	-0.14	-0.27	-0.21	-0.30	-0.41
	2	-0.37	-0.33	-0.40	-0.25	-0.32	-0.43
	3	-0.35	-0.36	-0.44	-0.25	-0.32	-0.41
Brain	1	-0.45	-0.68	-0.62	-0.56	-0.53	-0.56
	2	-0.59	-0.65	-0.58	-0.49	-0.50	-0.54
	3	-0.54	-0.57	-0.52	-0.44	-0.48	-0.54

1.2 Figures

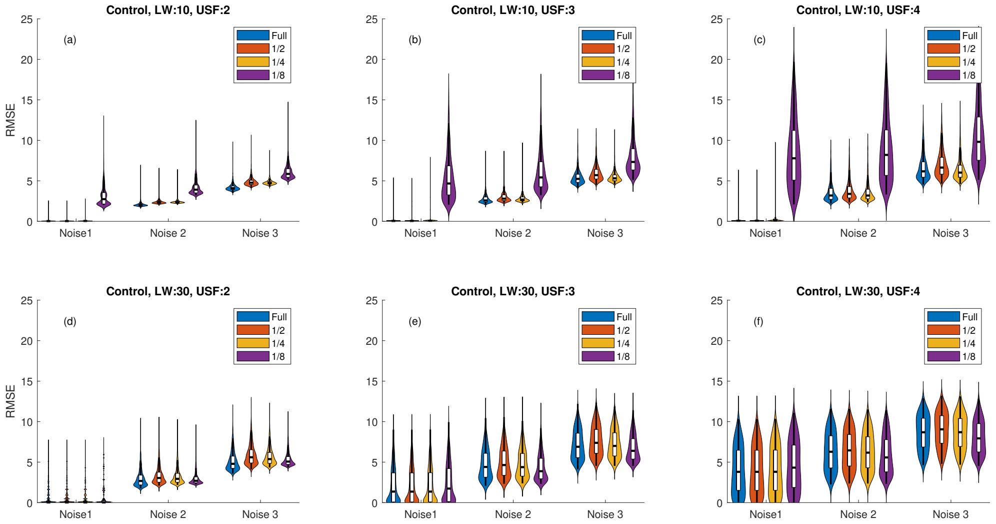

Figure S1. RMSE violin plots corresponding to 512-samples control simulation reconstructions with truncation. Each sub graph column corresponds to a different USF (x2, x3, x4) and sub graph rows to linewitdh of 10 and 30 Hz. USF of x2: using 256 out of 512. x3: using 170 out of 512. x4: using 128 out of 512.

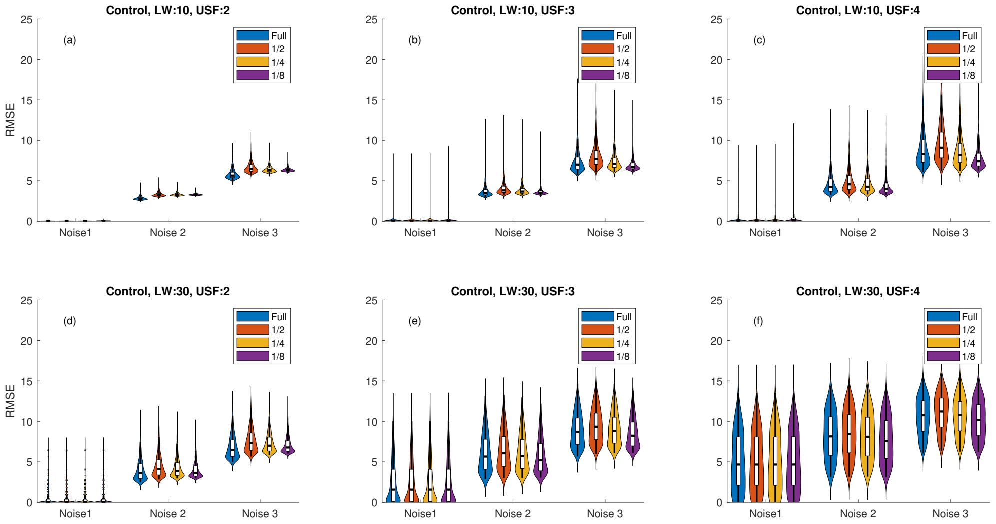

Figure S2. RMSE violin plots corresponding to 1024-samples control simulation reconstructions with truncation.Each sub graph column corresponds to a different USF (x2, x3, x4) and sub graph rows to linewitdh of 10 and 30 Hz. USF of x2: using 512 out of 1024. x3: using 341 out of 1024. x4: using 256 out of 1024.

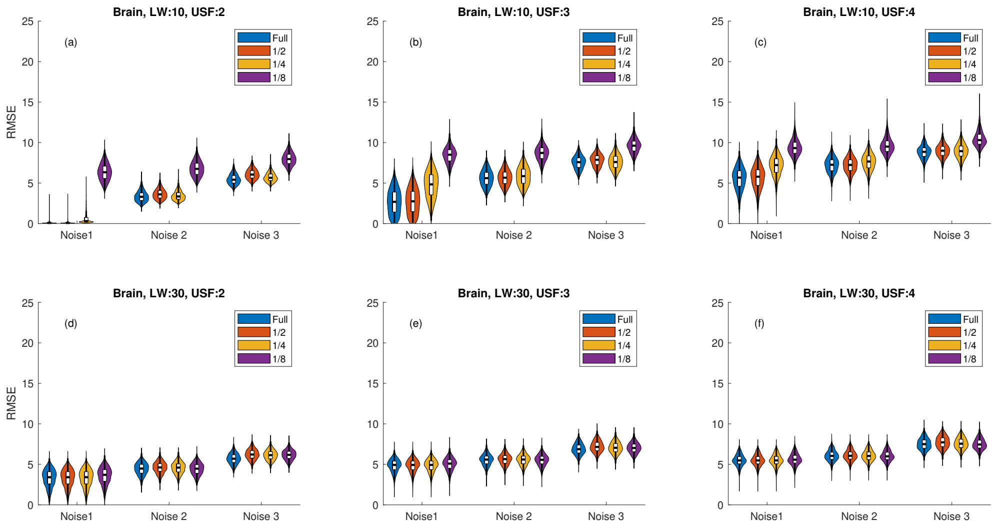

Figure S3. RMSE violin plots corresponding to 512-samples brain simulation reconstructions with truncation. Each sub graph column corresponds to a different USF (x2, x3, x4) and sub graph rows to linewitdh of 10 and 30 Hz. USF of x2: using 256 out of 512. x3: using 170 out of 512. x4: using 128 out of 512.

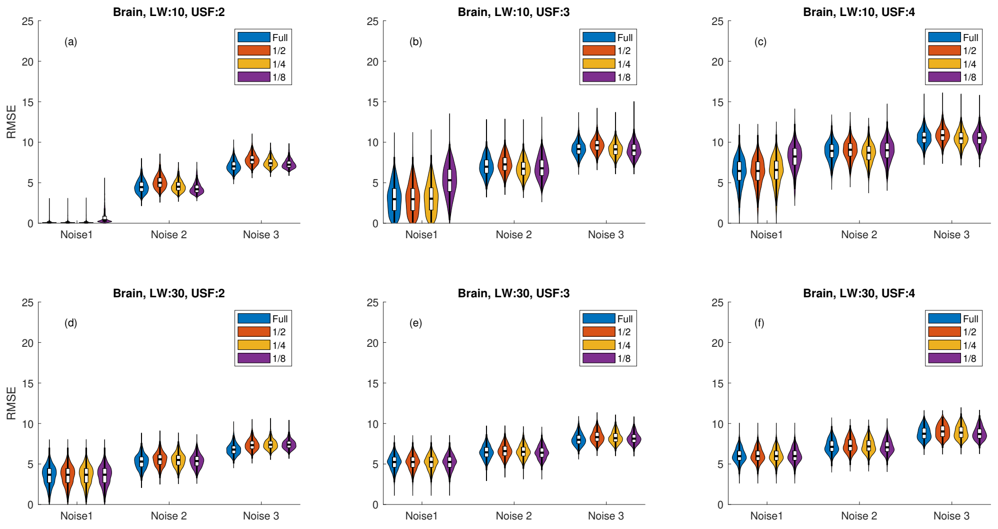

Figure S4. RMSE violin plots corresponding to 1024-samples brain simulation reconstructions with truncation. Each sub graph column corresponds to a different USF (x2, x3, x4) and sub graph rows to linewitdh of 10 and 30 Hz. USF of x2: using 512 out of 1024. x3: using 341 out of 1024. x4: using 256 out of 1024.

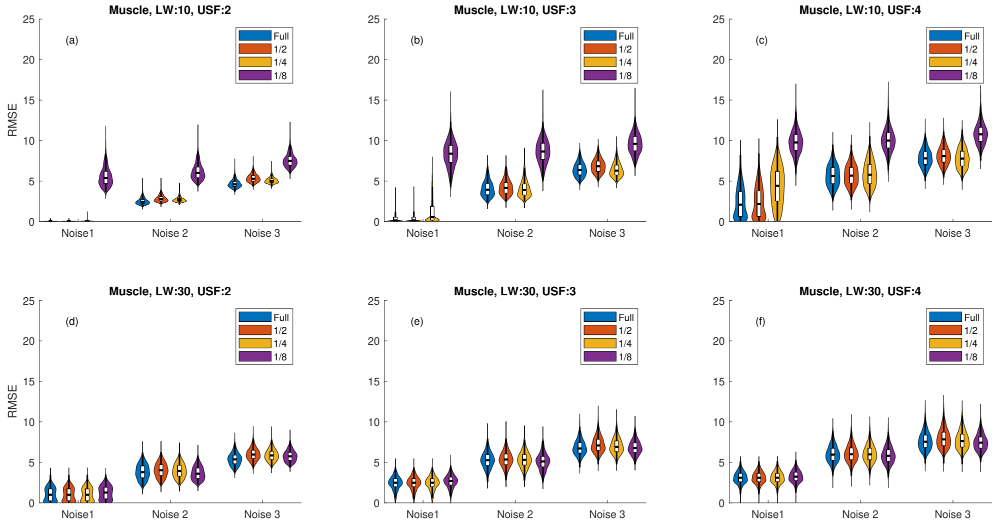

Figure S5. RMSE violin plots corresponding to 512-samples skeletal muscle simulation reconstructions with truncation. Each sub graph column corresponds to a different USF (x2, x3, x4) and sub graph rows to linewitdh of 10 and 30 Hz. USF of x2: using 256 out of 512. x3: using 170 out of 512. x4: using 128 out of 512.

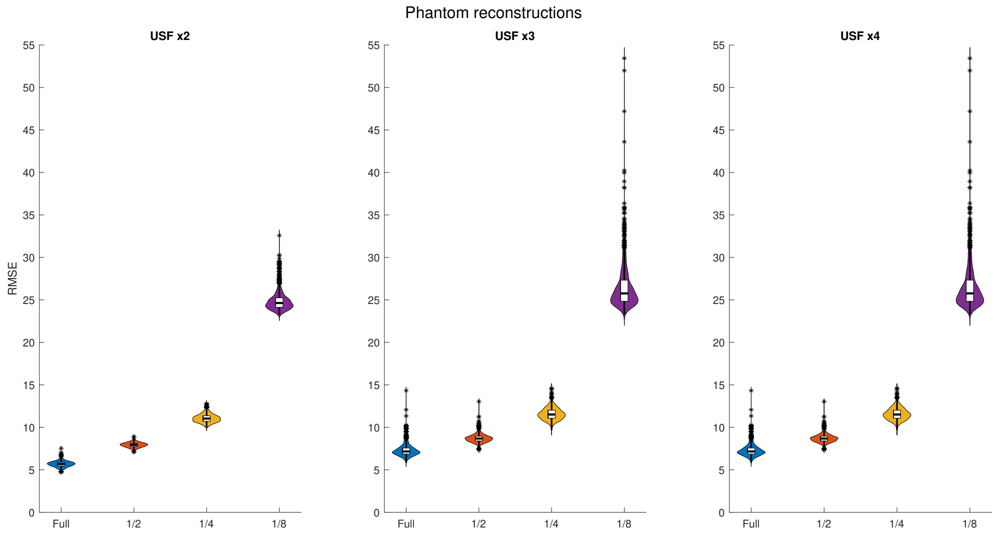

Figure S6. RMSE violin plots for phantom signal reconstructions, a clear increment in error distributions due to truncation is noticed. USF of x2: using 256 out of 512. x3: using 170 out of 512. x4: using 128 out of 512.

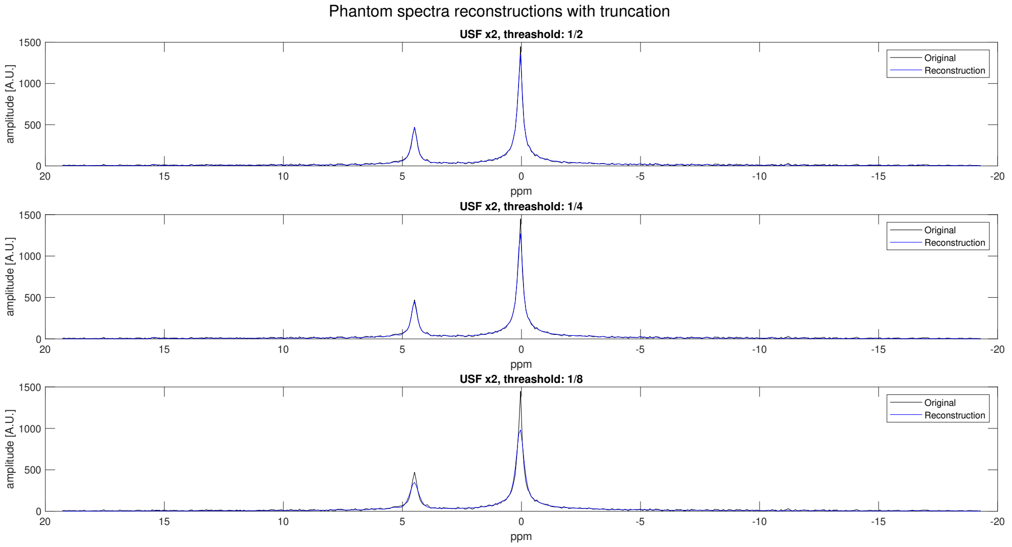

Figure S7. Examples of phantom spectra reconstructions with truncation. It is possible to observe the resolution loss in the compound peaks because of truncation experiments.

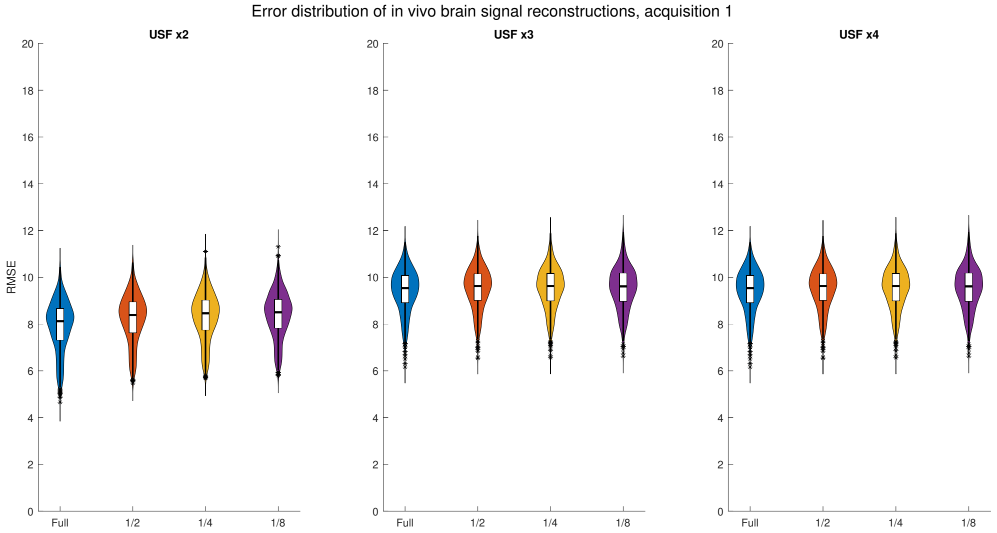

Figure S8. RMSE violin plots corresponding to in vivo brain signal reconstructions with truncation for the first acquisition. USF of x2: using 512 out of 1024. x3: using 341 out of 1024. x4: using 256 out of 1024.

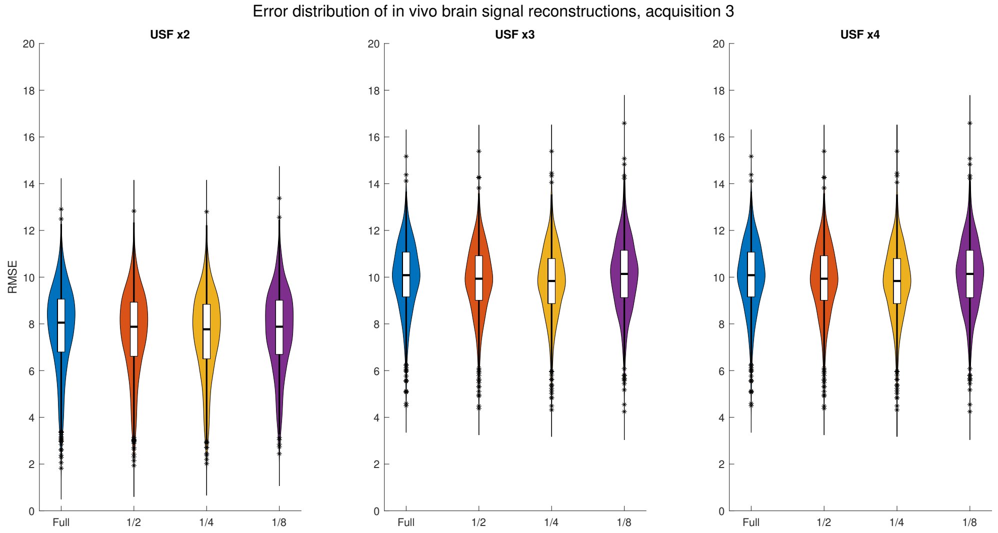

Figure S9. RMSE violin plots corresponding to in vivo brain signal reconstructions with truncation for the third acquisition. USF of x2: using 256 out of 512. x3: using 170 out of 512. x4: using 128 out of 512.

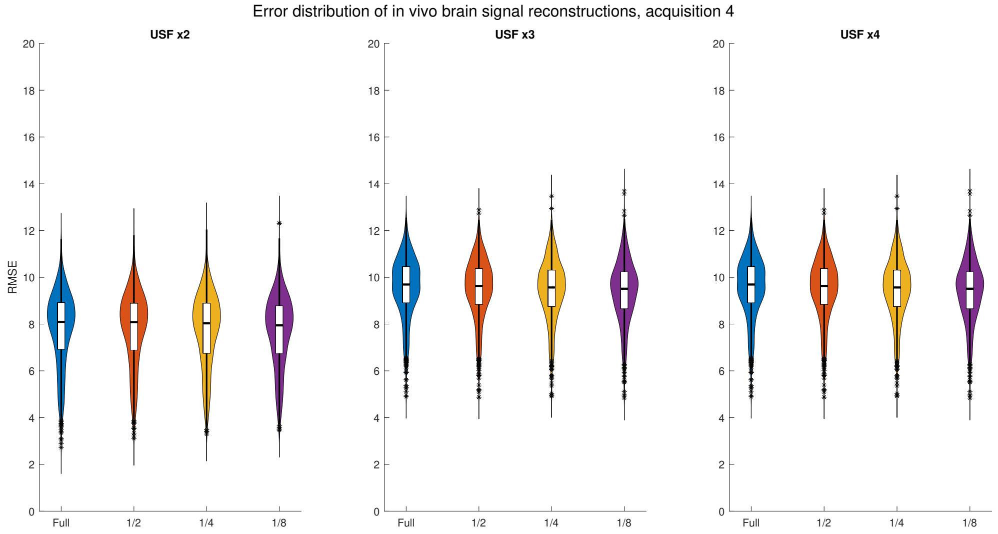

Figure S10. RMSE violin plots corresponding to in vivo brain signal reconstructions with truncation for the fourth acquisition. USF of x2: using 256 out of 512. x3: using 170 out of 512. x4: using 128 out of 512.

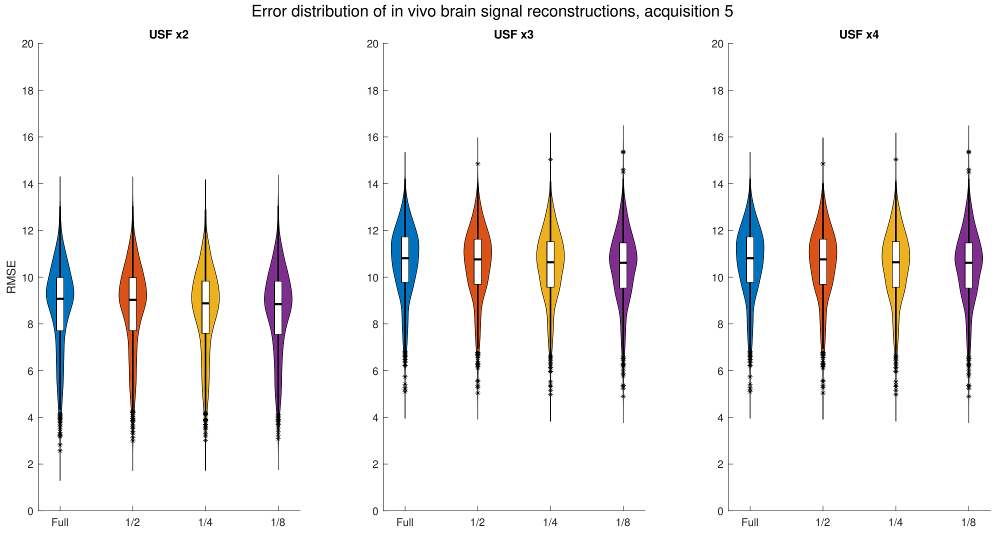

Figure S11. RMSE violin plots corresponding to in vivo brain signal reconstructions with truncation for the fifth acquisition. USF of x2: using 256 out of 512. x3: using 170 out of 512. x4: using 128 out of 512.

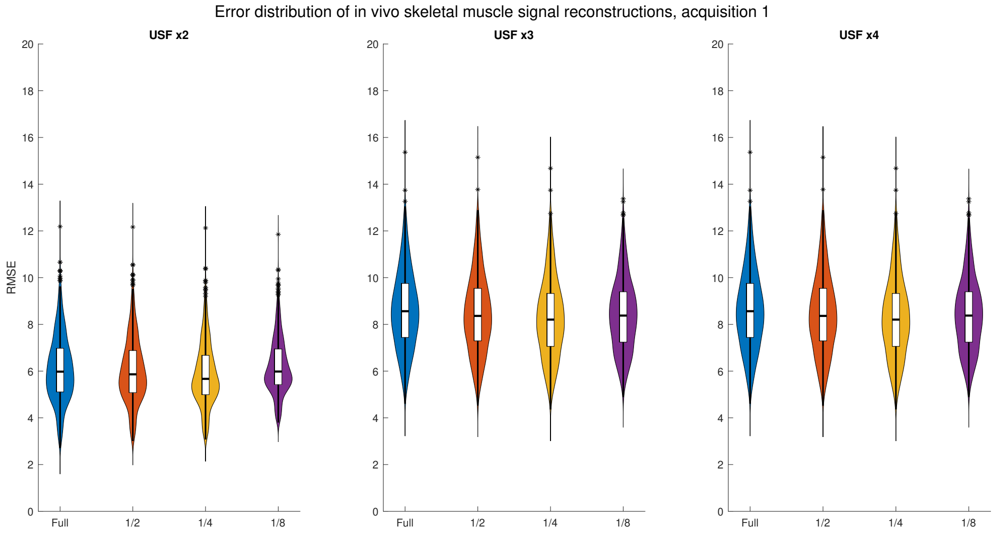

Figure S12. RMSE violin plots corresponding to in vivo skeletal muscle signal reconstructions with truncation for the first acquisition. USF of x2: using 512 out of 1024. x3: using 341 out of 1024. x4: using 256 out of 1024.

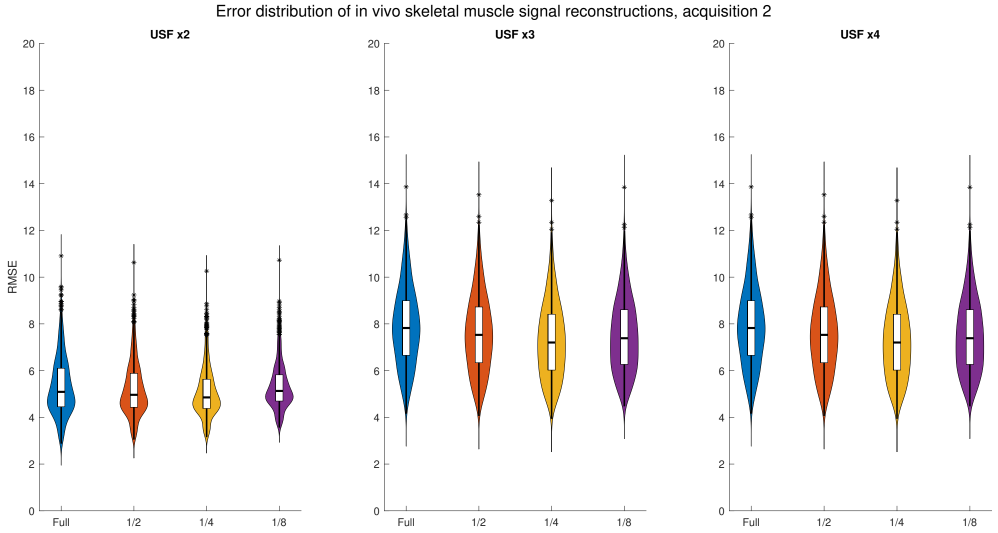

Figure S13. RMSE violin plots corresponding to in vivo skeletal muscle signal reconstructions with truncation for the second acquisition. USF of x2: using 512 out of 1024. x3: using 341 out of 1024. x4: using 256 out of 1024.

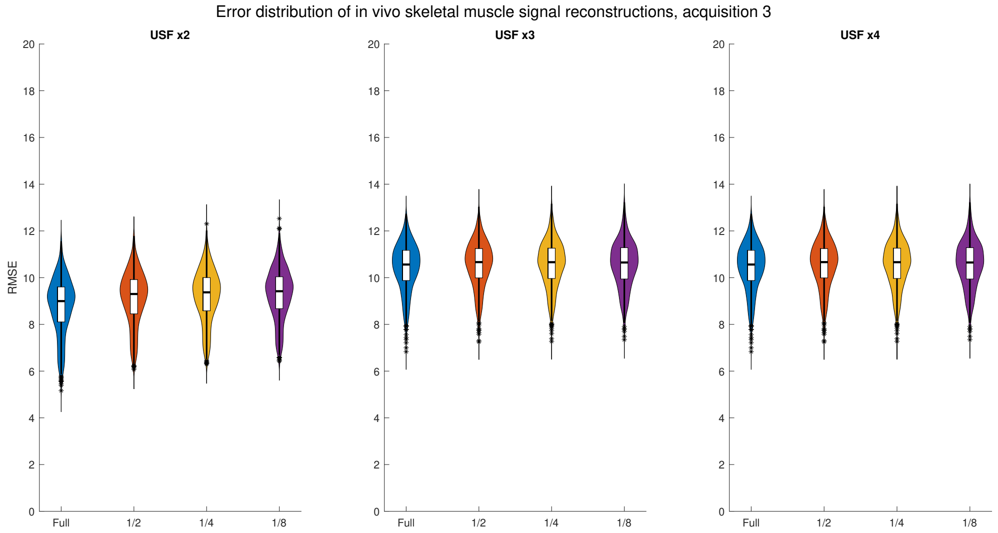

Figure S14. RMSE violin plots corresponding to in vivo skeletal muscle signal reconstructions with truncation for the third acquisition. USF of x2: using 512 out of 1024. x3: using 341 out of 1024. x4: using 256 out of 1024.

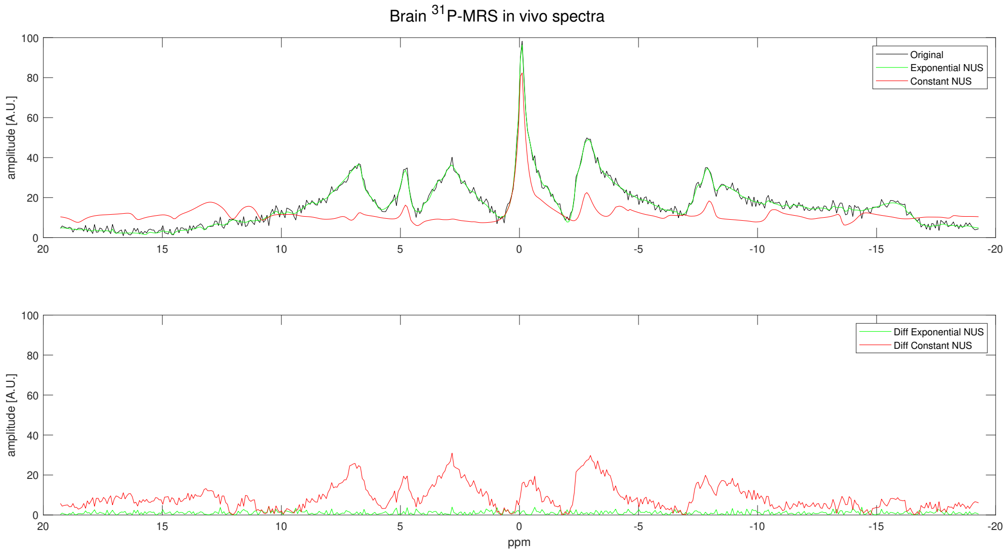

Figure S15. Examples in vivo ^{31}P MRS brain signal reconstruction using an exponentially NUS and constant NUS along with their differences. The original is the fully sampled (512 points) signal and both NUS patterns with USF x4: 120 out of 512 points.

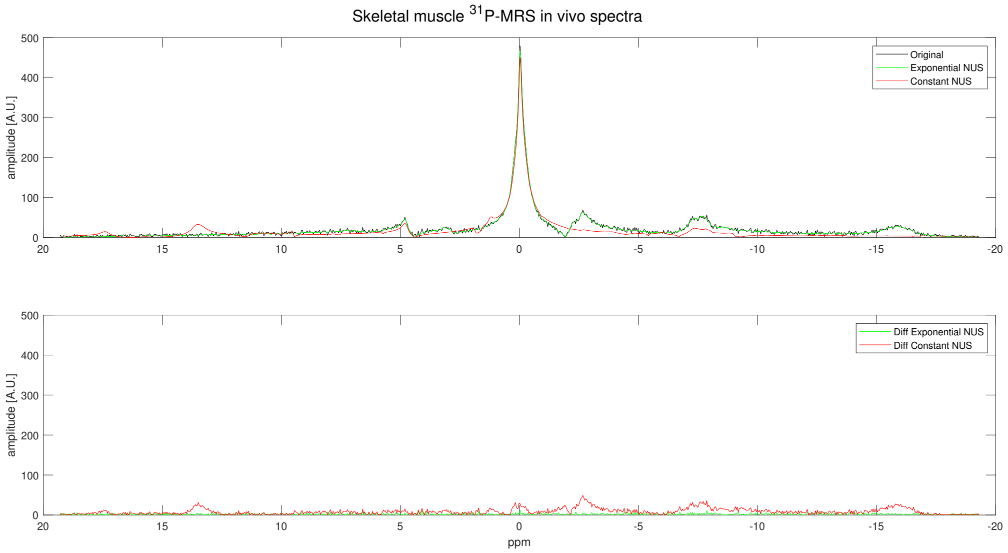

Figure S16. Examples in vivo ^{31}P MRS skeletal muscle signal reconstruction using an exponentially NUS and constant NUS along with their differences. The original is the fully sampled (512 points) signal and both NUS patterns with USF x4: 256 out of 1024 points.
